# Serological Conservation of Parasite-Infected Erythrocytes Predicts Plasmodium falciparum Erythrocyte Membrane Protein 1 Gene Expression but Not Severity of Childhood Malaria

**DOI:** 10.1128/IAI.00772-15

**Published:** 2016-04-22

**Authors:** George M. Warimwe, Abdirahman I. Abdi, Michelle Muthui, Gregory Fegan, Jennifer N. Musyoki, Kevin Marsh, Peter C. Bull

**Affiliations:** a Nuffield Department of Medicine, University of Oxford, Oxford, United Kingdom; b Centre for Research in Therapeutic Sciences and Institute for Healthcare Management, Strathmore University, Nairobi, Kenya; c Kenya Medical Research Institute-Wellcome Trust Research Programme, Kilifi, Kenya; d Department of Biochemistry and Chemistry, Pwani University, Kilifi, Kenya

## Abstract

Plasmodium falciparum erythrocyte membrane protein 1 (PfEMP1), expressed on P. falciparum-infected erythrocytes, is a major family of clonally variant targets of naturally acquired immunity to malaria. Previous studies have demonstrated that in areas where malaria is endemic, antibodies to infected erythrocytes from children with severe malaria tend to be more seroprevalent than antibodies to infected erythrocytes from children with nonsevere malaria. These data have led to a working hypothesis that PfEMP1 variants associated with parasite virulence are relatively conserved in structure. However, the longevity of such serologically conserved variants in the parasite population is unknown. Here, using infected erythrocytes from recently sampled clinical P. falciparum samples, we measured serological conservation using pools of antibodies in sera that had been sampled 10 to 12 years earlier. The serological conservation of infected erythrocytes strongly correlated with the expression of specific PfEMP1 subsets previously found to be associated with severe malaria. However, we found no association between serological conservation *per se* and disease severity within these data. This contrasts with the simple hypothesis that P. falciparum isolates with a serologically conserved group of PfEMP1 variants cause severe malaria. The data are instead consistent with periodic turnover of the immunodominant epitopes of PfEMP1 associated with severe malaria.

## INTRODUCTION

Malaria is still a major cause of childhood illness and death in Africa. However, repeated exposure to infection results in the acquisition of naturally acquired immunity (1), underpinning ongoing efforts to develop effective vaccines and other interventions. Immunity to severe malaria, which accounts for the bulk of deaths from malaria, develops relatively rapidly, while that to nonsevere disease may take many years to develop (1, 2). This suggests the existence of a relatively conserved set of targets of natural immunity among parasites causing severe disease. Support for this idea comes from observations that naturally acquired antibodies tend to recognize variant surface antigens (VSAs) on infected erythrocytes (IEs) from children with severe malaria more frequently than they recognize VSAs on IEs from children with nonsevere malaria (3–5), suggesting that parasite antigens on the IEs might account for the distinct rates of acquisition of immunity to these clinical manifestations. These commonly recognized, serologically conserved VSAs have been called VSA_FoRH_ (VSAs for which the frequency of recognition is high) (6) and VSA_SM_ (VSAs for severe malaria) (7).

Plasmodium falciparum erythrocyte membrane protein 1 (PfEMP1), an extremely diverse group of multidomain parasite molecules encoded by *var* genes (8), is thought to be the major parasite antigen on IEs and mediates IE binding to the host microvascular endothelium, resulting in parasite sequestration in host tissues and the multiorgan pathology associated with severe malaria. There is good evidence that PfEMP1 molecules are key targets of naturally acquired immunity to malaria (9, 10). However, PfEMP1 variants elicit a predominantly variant-specific antibody response (11), which, together with their extreme sequence diversity, raises the question of whether PfEMP1 molecules can underlie the relatively rapid acquisition of immunity to severe malaria. One model is that parasite virulence is linked to the ability of PfEMP1 molecules to adhere effectively to host cells and that this function in turn imposes constraints on antigenic diversity so that the best host cell binders are also the first to elicit host antibody responses. There is reasonable support for such an idea.

First, a subgroup of PfEMP1 molecules, called group A, was found in *in vitro* selection experiments to be well recognized by pools of IgG from malaria parasite-exposed children (7). The relative conservation of this group of PfEMP1 molecules is supported both through sequence analysis (12, 13) and through serological analysis of recombinant proteins (14). Second, group A PfEMP1 molecules have been found to be more commonly expressed by clinical isolates from children with severe malaria and low host immunity (15–19). Similar observations have recently been made for PfEMP1 molecules carrying a specific structural signature, or domain cassette (DC). PfEMP1 molecules carrying DC8 carry conserved epitopes and are associated with severe malaria (18, 20).

However, despite these data, it is still not clear whether severe malaria and VSA_SM_ (15) can be linked through a single set of serologically conserved PfEMP1 variants. This is because these previous studies have focused on either (i) crude serological associations of frequently recognized IEs with severe malaria (3–5), (ii) associations between specific PfEMP1 subgroups and seroprevalence (7, 14, 21, 22), or (iii) specific PfEMP1 subgroups and severe malaria (15–17, 19, 23–25). We therefore sought to test simultaneously the associations among the *var* gene transcription profiles of clinical parasite isolates from Kenyan children, the strength of recognition of erythrocytes infected by these parasites by sera from children in the same setting, and severe disease.

## MATERIALS AND METHODS

### Study participants and parasite sampling.

This work utilized a published *var* gene sequence data set (15), a quantitative PCR data set (27), and clinical isolates sampled from corresponding children presenting with malaria to Kilifi County Hospital in Kilifi, Kenya, between 2005 and 2007 (15). Sampling was done following the receipt of ethical approval from the Kenya Medical Research Institute Ethical Review Committee and informed consent from the study participants' parents or guardians (15, 19). Standard definitions of severe malaria based on parasitemia and clinical symptoms were used (15). Parasite cDNA was generated from uncultured ring-stage parasites sampled from children at the time of presentation to the hospital. This was used to generate *var* sequence data by classifying and counting the number of expressed sequence tags (ESTs) as described previously (15, 26) and by quantitative PCR using published primers and methods (18). For the EST approach, parasite cDNA was subcloned into bacterial cells, and up to 96 colonies of each parasite isolate were sequenced (15, 26). Following quality control of the sequence data, the number of individual colonies of each isolate carrying Cys2 and group A-like sequence types was expressed as a percentage and used for analysis (15, 26). Group A-like sequence types were defined here using a collection of polymorphic sequence blocks that distinguishes known group A PfEMP1 variants from non-group A PfEMP1 variants with high specificity and sensitivity (15). For the quantitative PCR approach, the transcript abundance of each *var* gene subset in each parasite isolate was determined and used for the analysis, as described previously (18). A published quantitative PCR data set was used for the analysis (27) and these attempts to measure the expression levels of (i) group A PfEMP1 (primers DBLa1 not var3, DBLa2/a1.1/2/4/7, and CIDRa1.6), (ii) DC8 PfEMP1 (primers CIDRa1.1, DBLa_CIDRa, DBLb12 and DBLb3/5, and DBLg4/6), (iii) DC13 and DC9 PfEMP1 (primers CIDRa1.4 and DBLz4, respectively), and (iv) group B and C PfEMP1 (primers B1 and C2, respectively) (17, 18).

### Measurement of the ability of IEs to be recognized by serum and plasma antibodies (serological conservation).

To determine the serological conservation of antigens expressed on IEs, we used sera from 800 healthy children aged 1 to 4 years sampled as part of a large cross-sectional survey conducted in 1995 (9, 28) and a set of parasite isolates sampled 10 to 12 years afterwards (2005 to 2007) (15). This ensured that our measure of serological conservation did not merely reflect the seroprevalence of antibodies to PfEMP1 variants that were commonly circulating at the time of sampling of the clinical parasite isolates. Four hundred of these serum samples were from children with detectable P. falciparum infection at the time of sampling, and the rest were from those without detectable parasites; all samples were evaluated for P. falciparum infection by light microscopy. To maximize the chances of detecting antibody specificities that were common in most children while diluting those that were rare, we made pools of sera for each of four age categories, that is, 1, 2, 3, and 4 years of age. For each age group, a pool of sera from 100 children without infection (parasite negative) was made and another pool of sera from 100 children with infection (parasite positive) was made. To identify parasite isolates expressing serologically conserved antigens, the reactivity of the IgG antibodies in these 8 pools against 92 parasite isolates from children with severe malaria (*n* = 47) or nonsevere malaria (*n* = 45) was tested by immunofluorescence assay (IFA) of live IEs quantified by flow cytometry.

The following additional assays were performed for comparison: (i) a subset of the parasites was tested for reactivity with these serum sample pools by an agglutination assay, and (ii) parasite isolates were tested for reactivity with a panel of 15 previously described plasma samples from children with acute malaria collected in the study setting between 1994 and 1996 by both IFA and an agglutination assay (3).

To assay the recognition of parasites by serum and plasma antibodies, ring-stage parasite isolates were cultured using standard methods to reach the mature trophozoite stage, when PfEMP1 is expressed on the IEs, and then cryopreserved in liquid nitrogen until the day of assay. To measure the strength of recognition of IEs by antibodies in the serum and plasma samples, IEs were thawed and adjusted to a maximum of 2% parasitemia using blood group O erythrocytes from a single donor. Total IgG binding to the IEs was then measured as described previously (19), and the mean fluorescence intensity (MFI) for each serum sample pool or plasma sample panel was acquired. All samples in the serum sample pools and the plasma sample panel were from individuals with blood group A (3, 28), which allowed assessment of IE recognition only for children with blood groups A and O. Agglutination assays were performed essentially as described previously (3), but with some modifications. Briefly, for each isolate, a 4% hematocrit of the thawed trophozoite-infected erythrocytes (which had been used for flow cytometry) was prepared in 11.5 μl phosphate-buffered saline containing 0.5% bovine serum albumin and 1 μl of test plasma in 96-well plates. Following rotation on a vertical rotor for 1 h, the cell suspension was transferred onto microscope slides, fixed with methanol, air dried, and stained with 5 μg/ml acridine orange. Scoring was done under a fluorescence microscope (Eclipse 80i; Nikon, Japan), and an agglutinate was defined as a clump of ≥5 intact trophozoite-infected erythrocytes. The number of agglutinates falling into each of four categories (size A, ≥5 to 25 IEs; size B, >25 to 125 IEs; size C, >125 to 625 IEs; size D, >625 IEs) was counted, and a conservative estimate of the number of IEs in agglutinates of each size category was determined by multiplying the total number of agglutinates in that size category by the lower limit of the number of IEs defining that size category. Thus, for each isolate, the number of size A clumps was multiplied by 5, the number of size B clumps was multiplied by 26, the number of size C clumps was multiplied by 126, and the number of size D clumps was multiplied by 626. The sum of the estimated number of IEs in the four size categories (here termed “agglutination score”) was then used for the analysis.

### Statistical analysis.

**FIG 1:**
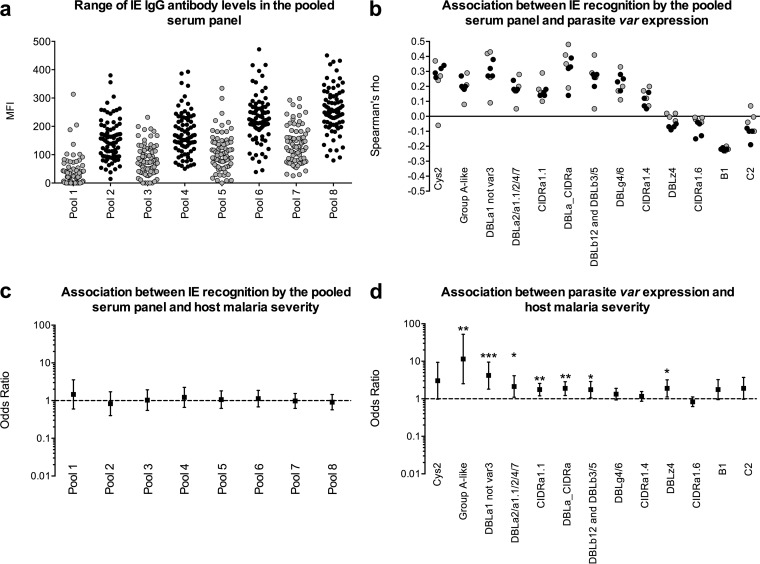
IE serological conservation correlates with parasite *var* gene expression profiles but not host malaria severity. Eight pools of serum samples, each composed of serum samples from 100 malaria parasite-exposed Kenyan children, were tested for IgG antibodies against each of 92 clinical isolates using IFA. (a) The mean fluorescence intensity (MFI) for each pool is shown. Pools 1, 3, 5, and 7 represent pools of serum samples from parasite-negative children aged 1, 2, 3, and 4 years, respectively, and MFIs are indicated as gray dots. Pools 2, 4, 6, and 8 represent pools of serum samples from parasite-positive children aged 1, 2, 3, and 4 years, respectively, and MFIs are indicated as black dots. (b) Spearman's rank correlation coefficients are shown for the relationship between the levels of parasite expression of various *var* gene subsets and recognition of the corresponding IEs by antibodies in each of the 8 serum sample pools. Gray and black dots, correlations calculated from antibodies from the parasite-negative and parasite-positive pools, respectively. The correlation coefficients and *P* values of the association between each *var* subset and responses in each serum sample pool are presented in Table S1 in the supplemental material. (c and d) Odds ratios, 95% confidence intervals, and *P* values (*, *P* < 0.05; **, *P* < 0.01; ***, *P* < 0.001) from unadjusted logistic regression models predicting the association of host disease severity with either the recognizability of parasites by the serum sample pools (levels of IgG antibody to IEs), where odds ratios are presented per 100 MFI units (c), or parasite *var* expression levels (d). In these models, recognition by each serum sample pool (c) or the expression levels of each *var* gene subset (d) are used in turn as continuous variables.

Stata software (v11) was used for all statistical analyses, and GraphPad Prism software (v6) was used for graphical representation of the data. Spearman's rank correlation coefficient was used to assess the relationship between *var* expression and the strength of recognition of IEs by antibodies in the serum sample pools (see Fig. 1b). Unadjusted logistic regression models were used to assess the associations between severe malaria and the strength of IE recognition (see Fig. 1c) and between severe malaria and *var* expression levels (see Fig. 1d). Each antibody or *var* expression measure was used to predict disease severity, and the odds ratios were plotted (see Fig. 1c and d). Disease severity was used as a binary variable, while both IE recognition (i.e., levels of IgG antibody to IE; see Fig. 1c) and *var* expression levels (see Fig. 1d) were used as continuous variables. In these analyses, quantitative PCR data were log transformed, whereas arcsine transformation was used for the EST analysis (15). Statistical significance was based on the conventional alpha value of 0.05, and the Bonferroni correction for multiple comparisons was performed where indicated. All the raw data used in the analyses are presented in Data Set S1 in the supplemental material.

## RESULTS AND DISCUSSION

Consistent with the findings of previous studies, the antibody responses in the serum sample pools measured using IFA were generally higher among parasite-positive children than children in whom the parasite was not detectable (28, 29) and increased with age (Fig. 1a). Overall, the proportion of the 92 isolates recognized by antibodies was greater for the pools of serum from parasite-positive children than the panel of 15 individual plasma samples from children presenting with nonsevere malaria (Fig. 1a; see also Fig. S1 in the supplemental material). A higher proportion of parasites was recognized by IFA than by agglutination (see Fig. S1 in the supplemental material). This supported the use of IFA detection of IgG in the serum sample pools to identify IE surface antigen epitopes that were present within the local parasite population both at the time of serum sampling and at the time of parasite sampling.

We related the recognition of the 92 parasite isolates by these serum sample pools to their corresponding *var* gene expression profiles using two approaches: (i) an expressed sequence tag (EST) approach that involved cloning and sequencing of PCR-amplified *var* sequence tags, which allows assessment of the relative number of group A-like genes expressed by classifying and counting the sequences amplified using a set of universal primers to the DBLα region of PfEMP1 (26, 30), and (ii) a quantitative PCR approach using a set of primers mostly directed toward PfEMP1 domain cassettes (DCs), including DC8 and DC13, that have previously been found to correlate with disease severity (18). Our expectation was that recognition of IE by antibodies in the serum sample pools, parasite *var* gene expression, and host disease severity would be correlated.

Expression of all *var* gene groups previously associated with severe malaria (15, 18, 19, 27) showed an association with the strength of recognition of IEs by at least one serum sample pool at the conventional cutoff for statistical significance (Spearman's rank correlation, *P* < 0.05) (Fig. 1b; see also Table S1 in the supplemental material). However, of these associations, only group A *var* expression, as measured by determination of Cys2 expression levels (15) and transcript abundance with primer DBLa1 not var3, and DC8 expression, as measured with primer DBLa_CIDRa and primers DBLb12 and DBLb3/5, met our threshold for statistical significance (Spearman's rank correlation, *P* < 0.0005) on the basis of a Bonferroni correction for the 104 comparisons performed (Fig. 1b; see also Table S1 in the supplemental material). Overall, this was consistent with previous evidence that antibodies from P. falciparum-exposed individuals frequently recognize group A and DC8 PfEMP1 variants (7, 14, 18, 21).

As previously reported from studies that used this data set, there were positive associations between severe malaria and expression of several of the *var* gene subsets considered here, with group A *var* expression showing the strongest associations (Fig. 1d) (15, 19, 27). However, in contrast to previous studies with antibodies sampled closer in time to the time of parasite sampling (3, 4, 6), we found no evidence for a significant difference between the ability of IEs causing severe malaria and those causing nonsevere malaria to be recognized by antibodies whether recognition by antibodies from serum sample pools (Fig. 1c) or in the panel of plasma samples was measured (see Data Set S1 in the supplemental material).

Where enough sample material was available (*n* = 59 samples, 29 of which were from children with severe malaria), we additionally typed the parasites by an agglutination assay, which was generally less sensitive than flow cytometry, and only two pools had detectable responses to >50% of the isolates (see Fig. S1 in the supplemental material). Nevertheless, the agglutination score showed positive associations with DC8 *var* subsets at the conventional level of statistical significance of a *P* value of <0.05, though these did not meet the cutoff for statistical significance after a Bonferroni adjustment for multiple comparisons was made (see Table S2 in the supplemental material). The agglutination score did not differ by disease severity whether it was measured in the panel of plasma samples or the serum sample pools (Mann-Whitney U test, *P* > 0.05 for all comparisons; see Data Set S1 in the supplemental material).

A plausible explanation for our observations is that whereas previous studies used a relatively short time interval of up to 3 years between the time of sampling of naturally acquired antibodies and the time of sampling of target IE, this study used a 10- to 12-year time interval. This raises the possibility that, while group A and DC8 *var* genes tend to be expressed mostly in immunologically naive individuals and the PfEMP1 variants expressed by these genes carry epitopes that are conserved over long periods, the variants that actually cause severe malaria may be prevalent over relatively short periods of a few years but then break apart through *var* gene recombination (31). This interpretation would emphasize an important role of immune evasion in parasite virulence. It should now be relatively straightforward to test this in studies specifically designed to explore antibody recognition of parasites causing severe malaria using different time periods between the time of collection of the sera and parasites.

In summary, by measuring *var* gene expression levels, we have found evidence that group A and DC8 PfEMP1 variants, whose bulk expression correlates with the severity of malaria, have restricted antigenic diversity. However, we found no evidence for an association between the serological conservation of IE antigens *per se* and severe malaria. These data contrast with the working hypothesis that severe malaria is caused by P. falciparum isolates with PfEMP1 variants whose immunodominant epitopes are maintained over long time periods. Further studies are needed to determine the rate of turnover within the local parasite population of PfEMP1 variants associated with severe malaria.

## Supplementary Material

Supplemental material
