# Supplementary material for: Serological Conservation of Parasite-Infected Erythrocytes Predicts Plasmodium falciparum Erythrocyte Membrane Protein 1 Gene Expression but Not Severity of Childhood Malaria
Source: Infect Immun. 2016 Apr 22;84(5):1331–5. doi: 10.1128/IAI.00772-15 (PMC4862716; doi:10.1128/IAI.00772-15)
Supplement: Supplemental material [file supp_84_5_1331__index.html]

Supplemental material 

# Serological Conservation of Parasite-Infected Erythrocytes Predicts Plasmodium falciparum Erythrocyte Membrane Protein 1 Gene Expression but Not Severity of Childhood Malaria

## Supplemental material

- Supplemental file 1 -

  Fig. S1. Comparison of IE antibody breadths in plasma and serum and their relationship with disease severity. Table S1. Correlations between *var* expression levels and IE recognition by serum pools by IFA. Table S2. Correlations between *var* expression levels and IE agglutination score by serum pools.

  PDF, 331K
- Supplemental file 2 -

  Dataset S1. Raw data used in the analyses.

  XLS, 64K
