# Supplementary material for: Serological Conservation of Parasite-Infected Erythrocytes Predicts Plasmodium falciparum Erythrocyte Membrane Protein 1 Gene Expression but Not Severity of Childhood Malaria
Source: Infect Immun. 2016 Apr 22;84(5):1331–5. doi: 10.1128/IAI.00772-15 (PMC4862716; doi:10.1128/IAI.00772-15)
Supplement: Supplemental material [file IAI.00772-15_zii999091673so1.pdf]

SUPPLEMENTAL MATERIAL

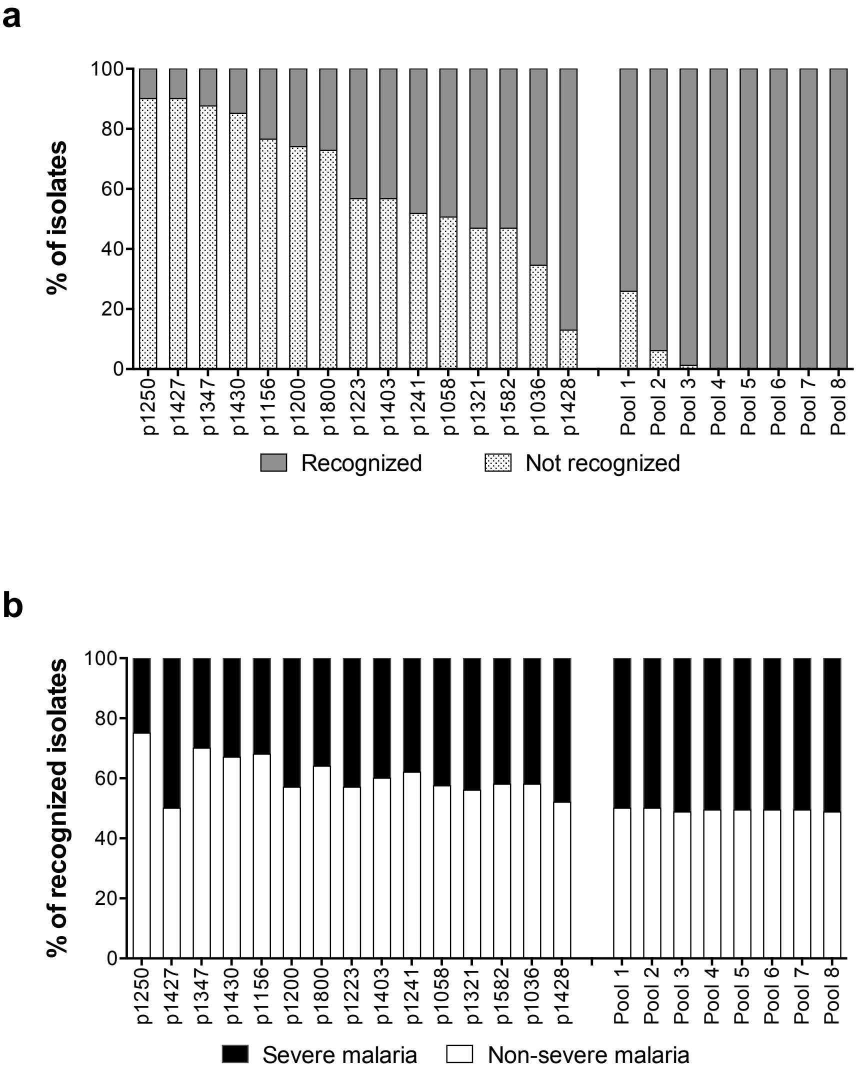

Figure S1: Comparison of IE antibody breadth in plasma and serum and their relationship with disease severity. The percentage of clinical isolates to which an antibody response is detected by flow cytometry in plasma (p1250 to p1428) and serum panels (pool 1 to pool 8) is shown in (a). Recognition is defined as mean fluorescence intensity of IgG binding >0 after subtracting background responses in plasma and sera from unexposed European donors. In (b) the percentage of isolates recognized in (a) are stratified by disease severity. The sample sizes for each parasite-plasma/serum pool pair are presented in Dataset S1.

***Var* gene subset**	**Pool 1 rho (*p*)**	**Pool 2 rho (*p*)**	**Pool 3 rho (*p*)**	**Pool 4 rho (*p*)**	**Pool 5 rho (*p*)**	**Pool 6 rho (*p*)**	**Pool 7 rho (*p*)**	**Pool 8 rho (*p*)**
Cys2	-0.06 (0.6)	0.32 (0.002)	0.24 (0.02)	0.26 (0.01)	0.27 (0.009)	0.29 (0.006)	**0.37 (0.0003)**	0.34 (0.001)
Group A-like	0.08 (0.4)	0.27 (0.009)	0.19 (0.07)	0.20 (0.05)	0.21 (0.04)	0.20 (0.05)	0.29 (0.004)	0.18 (0.1)
DBLa1 not var3	0.09 (0.5)	0.38 (0.0007)	0.26 (0.02)	0.32 (0.005)	**0.42 (0.0001)**	0.27 (0.02)	**0.42 (0.0001)**	0.27 (0.02)
DBLa2/a1.1/2/4/7	0.05 (0.7)	0.24 (0.04)	0.17 (0.1)	0.17 (0.1)	0.20 (0.09)	0.18 (0.1)	0.28 (0.01)	0.18 (0.1)
CIDRa1.1	0.1 (0.4)	0.18 (0.1)	0.18 (0.1)	0.14 (0.2)	0.16 (0.2)	0.16 (0.2)	0.29 (0.01)	0.14 (0.2)
DBLa_CIDRa	0.19 (0.1)	0.39 (0.0005)	0.35 (0.002)	0.33 (0.004)	**0.41 (0.0002)**	0.32 (0.005)	**0.48 (0.0001)**	0.29 (0.01)
DBLb12 and DBLb3/5	0.05 (0.7)	0.26 (0.02)	0.26 (0.02)	0.28 (0.01)	0.29 (0.02)	0.28 (0.01)	**0.42 (0.0002)**	0.20 (0.09)
DBLg4/6	0.11 (0.3)	0.28 (0.01)	0.17 (0.1)	0.17 (0.1)	0.2 (0.08)	0.23 (0.04)	0.33 (0.003)	0.25 (0.03)
CIDRa1.4	0.07 (0.6)	0.16 (0.2)	0.2 (0.08)	0.12 (0.3)	0.17 (0.1)	0.07 (0.6)	0.12 (0.3)	0.05 (0.7)
DBLz4	0.02 (0.9)	-0.05 (0.7)	0.02 (0.9)	-0.09 (0.4)	-0.005 (0.9)	-0.07 (0.5)	-0.03 (0.8)	-0.07 (0.5)
CIDRa1.6	-0.009 (0.9)	-0.05 (0.7)	-0.04 (0.7)	-0.04 (0.7)	-0.02 (0.9)	-0.12 (0.3)	-0.01 (0.9)	-0.15 (0.2)
B1	-0.20 (0.07)	-0.23 (0.04)	-0.23 (0.04)	-0.22 (0.05)	-0.22 (0.05)	-0.21 (0.06)	-0.22 (0.06)	-0.22 (0.06)
C2	0.07 (0.5)	-0.08 (0.5)	-0.004 (0.9)	-0.10 (0.4)	-0.04 (0.7)	-0.10 (0.4)	-0.10 (0.4)	-0.19 (0.09)

Table S1: Correlations between *var* expression levels and IE recognition by serum pools by IFA. The relationship between expression levels of each *var* gene subset and IgG antibody levels to IE is assessed using Spearman rank correlation coefficient (rho) and *p* values shown. The table represents a total of 104 comparisons. Associations remaining statistically significant after Bonferroni correction for multiple comparisons ($p<0.0005$) are shown in bold. See Figure 1b for a graphical representation of these data. Serum pools are as described in the text: Pool 1 – 1-year-old parasite negative children, Pool 2 – 1-year-old parasite positive children, Pool 3 – 2-year-old parasite negative children, Pool 4 – 2-year-old parasite positive children, Pool 5 – 3-year-old parasite negative children, Pool 6 – 3-year-old parasite positive children, Pool 7 – 4-year-old parasite negative children, Pool 8 – 4-year-old parasite positive children.

Var gene subset	**Pool 1** **rho (*p*)**	**Pool 2** **rho (*p*)**	**Pool 3** **rho (*p*)**	**Pool 4** **rho (*p*)**	**Pool 5** **rho (*p*)**	**Pool 6** **rho (*p*)**	**Pool 7** **rho (*p*)**	**Pool 8** **rho (*p*)**
Cys2	0.12 (0.4)	0.08 (0.5)	0.10 (0.5)	0.16 (0.2)	0.01 (0.9)	0.06 (0.6)	0.18 (0.2)	0.08 (0.5)
Group A-like	0.11 (0.4)	-0.01 (0.9)	-0.03 (0.8)	0.06 (0.6)	-0.07 (0.6)	-0.09 (0.5)	0.15 (0.3)	-0.06 (0.7)
DBLa1 not var3	0.17 (0.2)	0.03 (0.7)	0.03 (0.8)	0.11 (0.4)	-0.09 (0.5)	-0.10 (0.5)	-0.002 (0.9)	-0.16 (0.3)
DBLa2/a1.1/2/4/7	0.26 (0.06)	-0.12 (0.4)	-0.08 (0.6)	0.09 (0.5)	-0.10 (0.5)	-0.03 (0.8)	-0.05 (0.7)	-0.05 (0.7)
CIDRa1.1	0.40 (0.003)	0.10 (0.5)	0.25 (0.08)	0.22 (0.1)	0.11 (0.5)	0.09 (0.5)	0.25 (0.07)	0.08 (0.6)
DBLa_CIDRa	0.28 (0.05)	-0.01 (0.9)	0.16 (0.3)	0.12 (0.4)	0.002 (0.9)	0.001 (0.9)	0.06 (0.7)	-0.06 (0.7)
DBLb12 and DBLb3/5	0.43 (0.002)	-0.01 (0.9)	0.29 (0.04)	0.22 (0.1)	0.05 (0.7)	0.11 (0.4)	0.12 (0.4)	0.07 (0.6)
DBLg4/6	0.19 (0.2)	-0.07 (0.6)	-0.03 (0.8)	-0.05 (0.7)	-0.09 (0.5)	-0.19 (0.2)	-0.05 (0.7)	-0.19 (0.2)
CIDRa1.4	-0.16 (0.3)	-0.32 (0.02)	-0.18 (0.2)	-0.18 (0.2)	-0.16 (0.3)	-0.14 (0.3)	-0.29 (0.04)	-0.28 (0.05)
DBLz4	0.09 (0.5)	-0.26 (0.07)	0.15 (0.3)	-0.07 (0.6)	-0.20 (0.2)	-0.11 (0.4)	-0.02 (0.9)	-0.09 (0.5)
CIDRa1.6	0.18 (0.2)	-0.08 (0.6)	-0.10 (0.5)	0.01 (0.9)	0.004 (0.9)	-0.17 (0.2)	-0.20 (0.2)	-0.13 (0.4)
B1	-0.25 (0.08)	-0.28 (0.04)	-0.18 (0.2)	-0.33 (0.02)	-0.22 (0.1)	-0.27 (0.06)	-0.30 (0.03)	-0.28 (0.04)
C2	-0.02 (0.9)	-0.10 (0.5)	0.14 (0.3)	0.01 (0.9)	-0.04 (0.8)	0.03 (0.9)	0.04 (0.8)	-0.18 (0.2)

Table S2: Correlations between *var* expression levels and IE agglutination score by serum pools. The relationship between expression levels of each *var* gene subset and IE agglutination score is assessed using Spearman rank correlation coefficient (rho) and *p* values shown. The table represents a total of 104 comparisons. No associations remained statistically significant after Bonferroni correction for multiple comparisons ($p<0.0005$; compare with Table S2). Serum pools are as described in Table S1.
